# Photocatalytic Degradation of Quinolones by Magnetic MOFs Materials and Mechanism Study

**DOI:** 10.3390/molecules29102294

**Published:** 2024-05-13

**Authors:** Hongchao Chang, Guangyao Xu, Xiantong Huang, Wei Xu, Fujuan Luo, Jiarong Zang, Xiaowei Lin, Rong Huang, Hua Yu, Binbin Yu

**Affiliations:** 1 School of Pharmaceutical and Chemical Engineering, Taizhou University, Taizhou 318000, China; m19883610219@163.com (H.C.); m17726168964@163.com (G.X.); 13738903943@163.com (F.L.); zjr250442@outlook.com (J.Z.); 15860254463@163.com (X.L.); m17874492294@163.com (R.H.); 2 Ecological Environment Testing Centre, Zaozhuang 277300, China; zzhb2008@126.com; 3 Zhejiang Baima Lake Laboratory Co., Ltd., Hangzhou 310053, China; 21114078@zju.edu.cn; 4 Taizhou Biomedical and Chemistry Industry Institute, Taizhou 318000, China

**Keywords:** quinolones, magnetic MOFs, photocatalytic degradation, degradation mechanisms

## Abstract

With the rising incidence of various diseases in China and the constant development of the pharmaceutical industry, there is a growing demand for floxacin-type antibiotics. Due to the large-scale production and high cost of waste treatment, the parent drug and its metabolites constantly enter the water environment through domestic sewage, production wastewater, and other pathways. In recent years, the pollution of the aquatic environment by floxacin has become increasingly serious, making the technology to degrade floxacin in the aquatic environment a research hotspot in the field of environmental science. Metal–organic frameworks (MOFs), as a new type of porous material, have attracted much attention in recent years. In this paper, four photocatalytic materials, MIL-53(Fe), NH_2_-MIL-53(Fe), MIL-100(Fe), and g-C_3_N_4_, were synthesised and applied to the study of the removal of ofloxacin and enrofloxacin. Among them, the MIL-100(Fe) material exhibited the best photocatalytic effect. The degradation efficiency of ofloxacin reached 95.1% after 3 h under visible light, while enrofloxacin was basically completely degraded. The effects of different materials on the visible photocatalytic degradation of the floxacin were investigated. Furthermore, the photocatalytic mechanism of enrofloxacin and ofloxacin was revealed by the use of three trappers (▪O_2_^−^, h^+^, and ▪OH), demonstrating that the role of ▪O_2_^−^ promoted the degradation effect of the materials under photocatalysis.

## 1. Introduction

New pollutants, including persistent organic pollutants, antibiotics, endocrine disruptors, and microplastics were biotoxic, environmentally persistent, and bioaccumulative with great risk to the ecological environment or human health [1]. Compared with traditional pollutants, new pollutants typically consisted of composite or mixed substances rather than single substances. They are also characterised by a complex structure, high stability, bioaccumulation, and persistence, and long degradation cycles. Therefore, it is difficult to degrade them completely using traditional methods such as physical adsorption and sedimentation [2]. New methods of pollutant treatment are urgently required for the protection of environment and human health [3].

Among various pollutants, quinolone is a widely used antibiotic due to its excellent bactericidal effect in clinical settings. However, antibiotics sewage discharged into the ecological environment can migrate to agricultural and sideline products, induce biological resistance, and affect plants, animals, and human health [4,5,6,7]. Nowadays, the detection of drug resistance in animal and human pathogens has become a common phenomenon. According to the analytical testing, the quinolone content in livestock manure soil samples was generally higher than the control limit of 49.77 μg/kg [8,9]. Moreover, the average contents of norfloxacin (QNC) and ofloxacin (OFL) in certain greenhouse soil samples could be as high as 373.73 μg/kg and 643.34 μg/kg, respectively, significantly exceeding the trigger value (100 μg/kg) for the ecotoxicological effect of antibiotics. For example, in an important vegetable growing area in Shandong Province, China, quinolone residues were found with a maximum ofloxacin concentration of 0.288 mg/kg [10]. The ecological degradation of new antibiotic drugs into the biological environmental system is often slow. Although we have stopped promoting their use, there is still a significant amount of accumulation in the global ecology [11]. If timely and effective measures are not taken, floxacin compounds will accumulate in large quantities, potentially leading to impacts on the global ecosystem and human health issues.

Nowadays, with the attention paid to the issue of antibiotic contamination, many studies for the degradation of quinolone antibiotics have emerged. The traditional methods of artificial degradation are mainly biodegradation and chemical treatment. Biodegradation were aerobic method, anaerobic method, and their combination. Chemical treatment mainly used strong oxidation to destroy the molecular structure. Chemical degradation process was mainly conducted with Fenton reagent, O_3_, O_3_/UV, UV/H_2_O_2_ and so on [12,13]. However, these conventional methods have some drawbacks, such as harsh operating conditions, inadequate performance, and susceptibility to secondary pollutants. Photocatalysis, as one of the advanced oxidation processes (AOPs), has progressed rapidly since the discovery of the photoelectrochemical water splitting reaction using a semiconductor by Fujishima and Honda in 1972. It is considered an environmentally friendly, sustainable, and energy-efficient technology. The technology can be applied in cases of low biodegradability, high complexity, and a high concentration of pollutants in wastewater [14]. Photocatalysts are responsible for utilising solar energy to degrade pollutants and make the wastewater treatment process economically viable [15]. Thus, the photocatalytic material technology is widely used due to many advantages such as safety, efficiency, and sustainability [16,17,18].

MOFs had high porosity, low density, high specific surface area, adjustable pore channels, and a rich topological structure [19,20,21]. It could be widely used in heavy metal recovery, gas adsorption, photocatalysis, and other fields. The Lavoisier skeleton (MIL) series of materials was a kind of porous metal carboxylate. Among them, MIL-Fe MOFs not only inherit several characteristics from conventional MOF materials, such as a large pore volume and a high specific surface area, but they also possess abundant Lewis acid centres, Brønsted acid centres, and unsaturated metal sites, making them exceptionally well suited for photocatalytic applications. For example, Oveisi et al. combined MIL-100 (Fe) and inorganic nanofibers for the visible light degradation of methylene blue (MB) [22]. Li et al. covalently modified NH_2_-MIL-101 (Fe) with 2-anthraquinone sulfonic acid (AQS) and used it as a redox mediator to augment the degradation of bisphenol A through persulfate activation. The result showed that AQS NH_2_-MIL-101 (Fe) led to an impressive 97.7% degradation of BPA [23]. Cao et al. reported MIL-53(Fe)/PS/LED visible light photocatalytic degradation system for Acid Orange, the degradation rate reached an impressive 100% [24]. However, there have been few reports on the application of MIL-Fe MOFs in the photodegradation of quinolone pollutants, as well as the exploration of their degradation mechanisms. Also, the photocatalytic degradation mechanisms of these MOFs on quinolines were not compared in detail. Thus, it is very necessary to compare the photocatalytic degradation of quinoline for these MIL-Fe MOFs, and further analyse the differences in the inherent photocatalytic mechanism.

In this work, three MIL materials, MIL-53(Fe), NH_2_-MIL-53(Fe), MIL-100(Fe), and a classic photocatalytic material, g-C_3_N_4_, were synthesised to carry out the visible-light-catalysed removal of ofloxacin and enrofloxacin. Among them, the MIL-100(Fe) material exhibited the best photocatalytic effect. The degradation efficiency of ofloxacin reached 95.1% after 3 h under visible light, while enrofloxacin was basically completely degraded. The effects of different materials on the visible photocatalytic degradation of floxacin were also investigated. Furthermore, the photocatalytic mechanism of enrofloxacin and ofloxacin was revealed by the use of three trappers (▪O_2_^−^, h^+^, and ▪OH), demonstrating that the role of ▪O_2_^−^ promoted the degradation effect of the materials under photocatalysis. It provides important theoretical significance for the photocatalytic application of MOF-based materials.

## 2. Results and Discussion

### 2.1. Characterisations

#### 2.1.1. SEM Analysis

**Figure 1:**
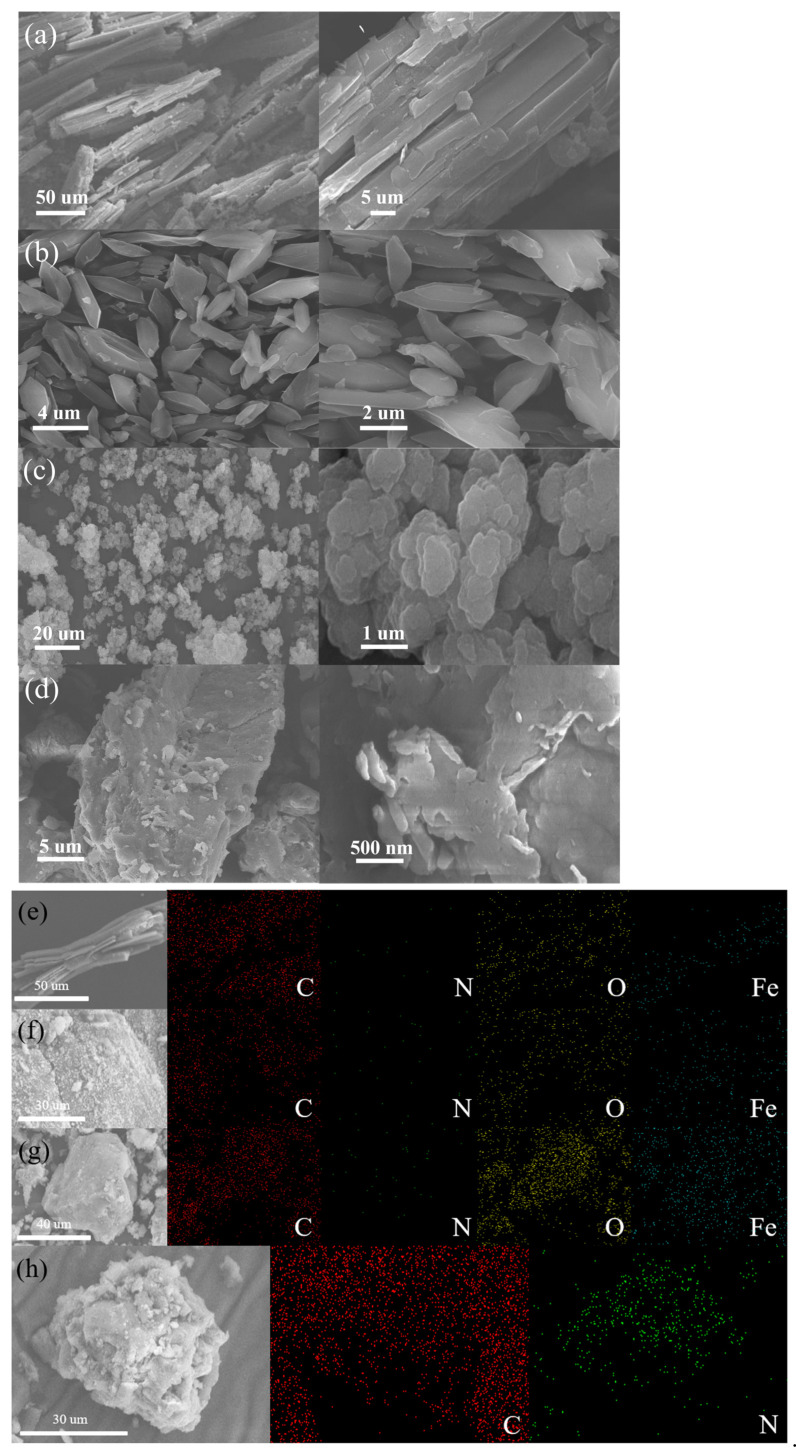
SEM images of (**a**) MIL-53(Fe), (**b**) NH_2_-MIL-53(Fe), (**c**) MIL-100(Fe), (**d**) g-C_3_N_4_ and EDX elemental mapping of (**e**) MIL-53(Fe), (**f**) NH_2_-MIL-53(Fe), (**g**) MIL-100(Fe), and (**h**) g-C_3_N_4_.

Figure 1 showed the SEM images of four photocatalytic materials, namely MIL-53(Fe), NH_2_-MIL-53(Fe), MIL-100(Fe), and g-C_3_N_4_. As shown in Figure 1a, the MIL-53(Fe) material was in the shape of a rod structure with multiple small spherical protruding particles attached to the surface. The MIL-53(Fe) size was about 100 µm. Figure 1b showed that the NH_2_-MIL-53(Fe) material was dispersed with the form of rhombic blocks, its structure was significantly different from that of MIL-53(Fe), which may be due to the introduction of 2-aminoterephthalic acid ligand. Compared with MIL-53(Fe), a smaller size of about 2–5 µm and a smoother surface were observed on NH_2_-MIL-53(Fe). Figure 1c showed that the MIL-100(Fe) material exhibited the petal-like shape with the state of layer-by-layer stacking. In Figure 1d, g-C_3_N_4_ could be seen as a block of layered structures. As shown in Figure 1e–g, the EDX elemental mapping images of MIL-53(Fe), NH_2_-MIL-53(Fe) and MIL-100(Fe) exhibited the existence and spatial distributions of Fe, C, N, O species within the whole framework. The EDS elemental mapping image of g-C_3_N_4_ is distinctly differed from the iron-containing frameworks, Figure 1h showed the existence and spatial distributions for C and N, and consisted with its graphitic carbon nitride structure.

#### 2.1.2. XRD Analysis

**Figure 2:**
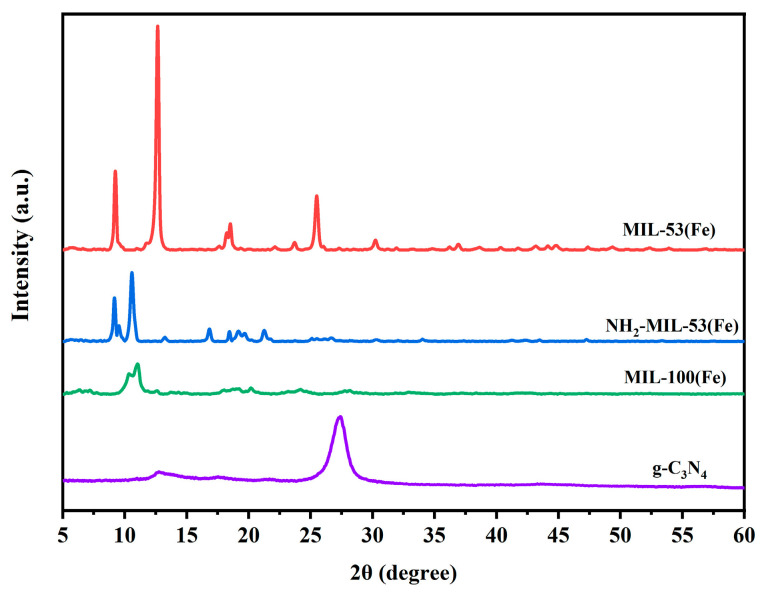
XRD patterns of MIL-53(Fe), NH_2_-MIL-53(Fe), MIL-100(Fe), and g-C_3_N_4_.

The XRD analysis was performed to further investigate the crystal structure of MIL-53(Fe), NH_2_-MIL-53(Fe), MIL-100(Fe), and g-C_3_N_4_. As can be seen from Figure 2, the strong diffraction peaks appear in MIL-53(Fe) at 2θ degrees of about 9.4°, 12.6°, 17.8°, 25.4°, and 27.3°. This was similar to the previous literature report [25]. NH_2_-MIL-53(Fe) showed characteristic diffraction peaks at 2θ degrees of 8.9°, 10.1°, 14.7°, 16.7°, 17.8°, 24.1°, and 25.9°. These were similar to the previous literature reports [26]. These also verify the successful synthesis of the material. Comparing the images of MIL-53(Fe) and NH_2_-MIL-53(Fe), there was a significant difference in the peak positions and intensity. This showed that different ligands also affect the structure of the MOF materials. The characteristic peaks of MIL-100(Fe) mainly appeared at 2θ degrees of 10.2° and 11.0° [27], and the characteristic peaks of the g-C_3_N_4_ were around 27.5°, which correspond to the crystalline diffraction peaks of raw g-C_3_N_4_ [28].

#### 2.1.3. FT-IR Analysis

**Figure 3:**
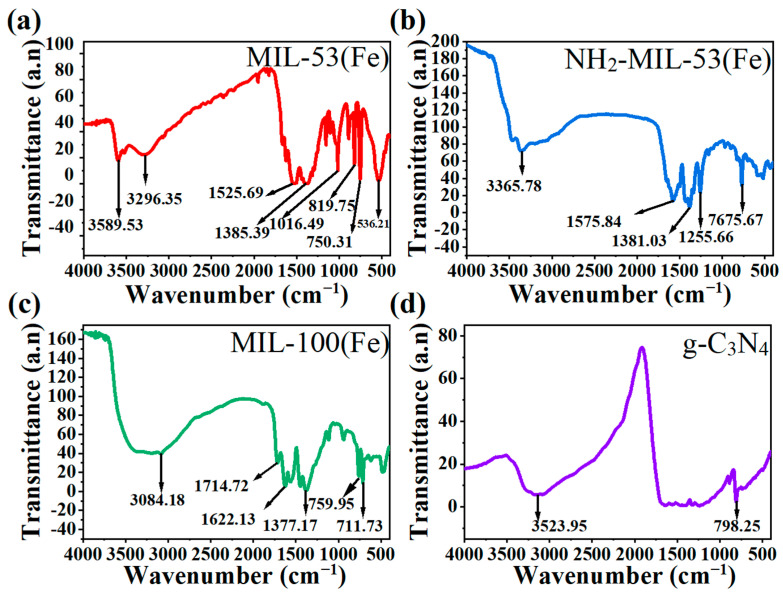
FT-IR spectra of (**a**) MIL-53(Fe), (**b**) NH_2_-MIL-53(Fe), (**c**) MIL-100(Fe), and (**d**) g-C_3_N_4_.

As shown in the FT-IR spectra, Figure 3a–c all had free carboxylic acid C=O bond stretching vibration peaks around 1700–1750 cm^−1^ and a benzene ring backbone vibration peaks at 1450–1650 cm^−1^. The difference was that Figure 3a had a single peak with a para-substitution of the benzene ring at 780–860 cm^−1^ [29]. Figure 3b had a peak of –NH_2_ vibrational at 3365.7 cm^−1^. Figure 3c had two peaks at around 750–800 cm^−1^ and 700 cm^−1^, which correspond to the tri-substituted peaks of benzene ring 1,3,5. They correspond to terephthalic acid, 2-aminoterephthalic acid in the synthetic raw materials, respectively. Figure 3d had –NH_2_ bond vibration peak at 3523.95 cm^−1^ and triazine ring vibration peak at 798.25 cm^−1^. This corresponds to the raw material melamine.

#### 2.1.4. XPS Analysis

**Figure 4:**
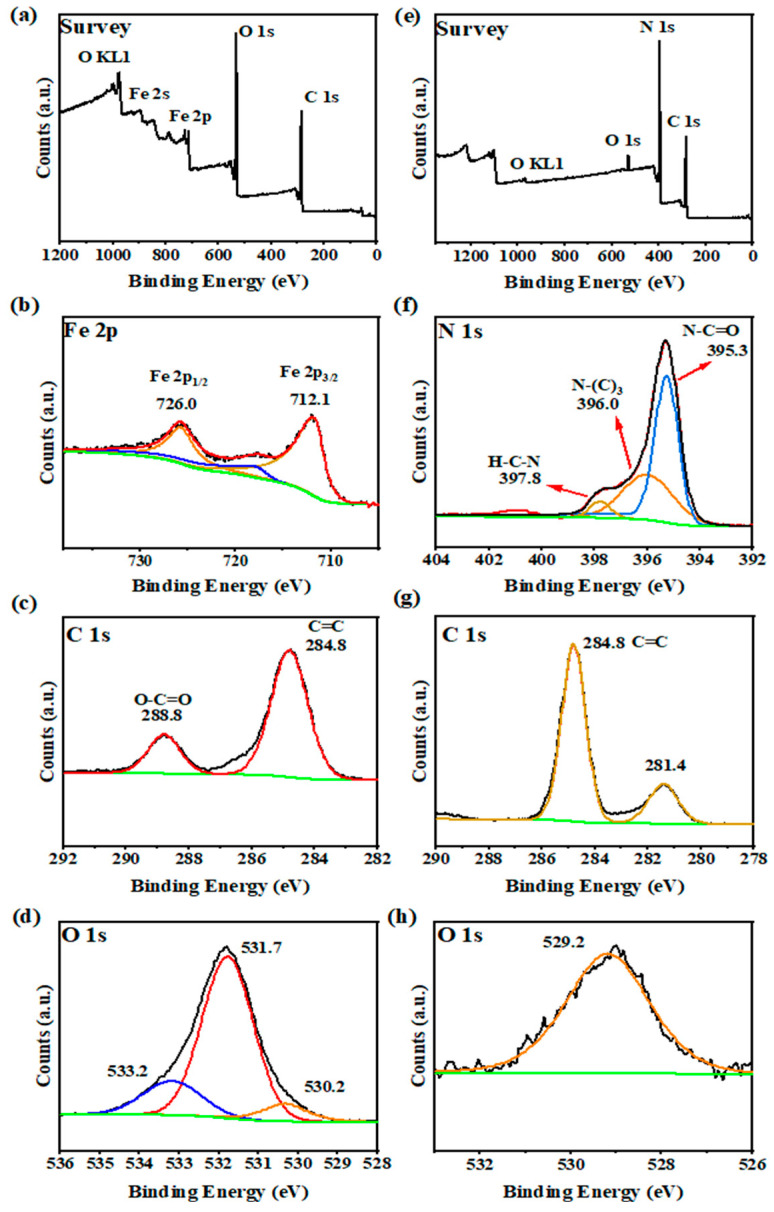
XPS spectra of MIL-100(Fe): (**a**) Survey, (**b**) Fe 2p, (**c**) C 1s, (**d**) O 1s and g-C_3_N_4_: (**e**) Survey, (**f**) N 1s, (**g**) C 1s, (**h**) O 1s.

We added the XPS measurements of MIL-100(Fe) and g-C_3_N_4_, and the results are shown in Figure 4. The survey spectra (Figure 4a) indicate the presence of Fe, O, and C elements in MIL-100(Fe). For the Fe 2p spectrum (see Figure 4b), the binding energy peak near 712.1 eV is attributed to Fe 2p_3/2_, and the peak near 726.0 eV is attributed to Fe 2p_1/2_. The XPS spectrum of C 1s (Figure 4c) showed two peaks at 284.8 and 288.8 eV. The peaks at 284.8 and 288.8 eV correspond to phenyl (C–C/H) and carboxyl (C=O) signals, respectively. In the O 1s spectrum of MIL-100(Fe) (Figure 4d), three peaks located at 530.2, 531.7, and 533.2 eV are related to O in the iron oxide (Fe–O–Fe), iron bonded to hydroxyl/organic ligands (FeO–C), and unbonded carboxyl (O–C=O), respectively [30]. These are corresponding to the reported MIL-100(Fe), and all these results clearly confirm the formation of the MIL-100(Fe) photocatalyst.

The g-C_3_N_4_ XPS spectrum (Figure 4e) shows the presence of C, O, and N elements. For N 1s (Figure 4f), there are three peaks corresponding to hetero nitrogen (N_2_C, 395.3 eV), tertiary nitrogen (N_3_C, 396.0 eV), and an amino group (NH_2_, 397.8 eV) within the aromatic ring. The XPS spectrum of C 1s (Figure 4g) showed two peaks at 281.4 and 284.8 eV. For O 1s (Figure 4h), there was only one peak. Based on the above discussion, g-C_3_N_4_ was successfully obtained [31].

#### 2.1.5. BET Analysis

**Figure 5:**
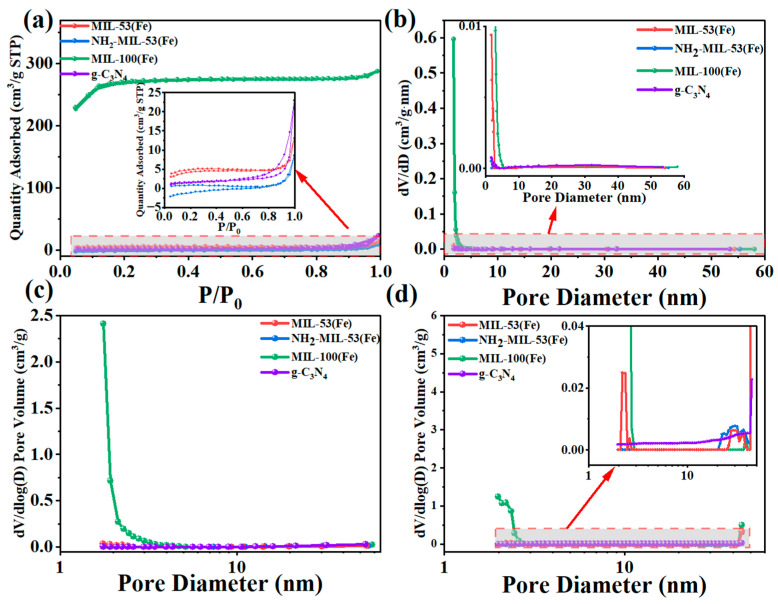
(**a**) N_2_ adsorption–desorption isotherms at 77 K, (**b**) BJH, (**c**) HK pore volume distribution and (**d**) DFT pore size distribution curves of MIL-53(Fe), NH_2_-MIL-53(Fe), MIL-100(Fe), and g-C_3_N_4_.

The BET specific surface area results are shown in Table 1. It could be seen that the specific surface area of MIL-100(Fe) was the largest reaching 900.15 m^2^/g, with an average pore size of 1.98 nm. The specific surface area of NH_2_-MIL-53(Fe) was the smallest, only 2.81 m^2^/g. As shown in Figure 5a, these isotherms were of mixed types (i.e., isotherms I, II, and IV) in the IUPAC classification [32]. The isotherm of g-C_3_N_4_ belonged to the typical type IV isotherm. It appeared as an adsorption hysteresis backline. The initial portion of the other MIL-100(Fe) isotherms was of type I, with faster adsorption at low relative pressures. It corresponded to N_2_ adsorption in the micropores. At moderate and higher relative pressures, the products showed type II isotherms with a slight increase in adsorption but no saturation of adsorption. NH_2_-MIL-53(Fe) and MIL-53(Fe) basically showed type II isotherms with the least amount of adsorption. Pore size distribution (PSD) curves, specifically dV/dD and dV/dlog (D) (V was the pore volume) versus D (D was the pore diameter), were widely used to analyse pore size distributions and to compare the contribution of different pore size ranges to the total pore volume [33]. We used the BJH model (Figure 5b), the Horvath–Kawazoe (HK) model (Figure 5c), and the density-functional theory (DFT) model (Figure 5d) to investigate the distribution of mesopore, micropore, and total pore sizes, respectively. In Figure 5b, the curves for all three samples except MIL-100(Fe) showed a gradual increase from mesopores to smaller pores, while MIL-100(Fe) showed a sharp decrease. As could be seen from Figure 5c, the main micropores were small in size, with peaks around 2 nm for MIL-100(Fe). Combined with the DFT model (Figure 5d), MIL-100(Fe) also had only some micropores and mesopores around 2 nm–3 nm. The results suggested that MIL-100 had a higher specific surface area and microporous structure. This could provide more surface active sites and make it easier for charge transfer through the catalyst substance, and thus enhanced the photocatalytic activity.

### 2.2. Optical Characteristic Analysis

**Figure 6:**
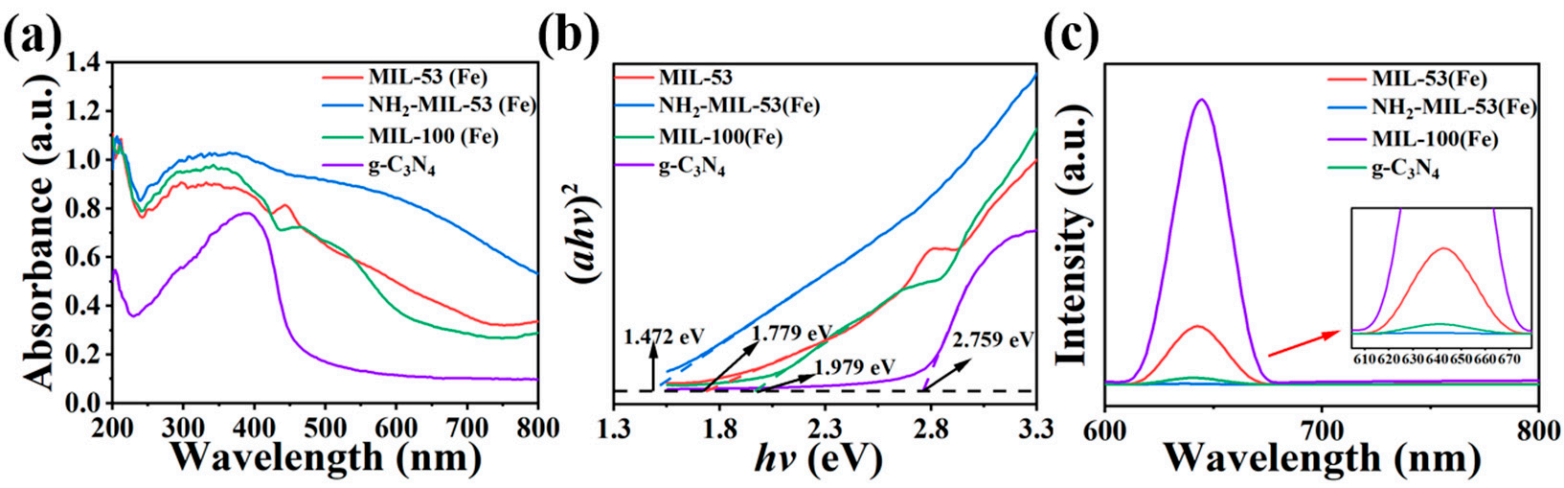
(**a**) UV diffuse reflectance pattern, (**b**) forbidden band calculations, and (**c**) fluorescence spectra of MIL-53(Fe), NH_2_-MIL-53(Fe), MIL-100(Fe), and g-C_3_N_4_.

As shown in Figure 6a,b, the strongest absorption bands of the synthesised materials were all below 450 nm, which suggested that these materials were more capable of absorbing UV light. The optical data of the four prepared samples were analysed near the absorption edge according to the formula (*αhν*)^2^ = *A*(*hν* − *E_g_*), where *α* was the absorption coefficient, *ν* was the optical frequency, *A* was the absorption constant, *h* was Planck’s constant, and *E_g_* was the forbidden bandwidth. Using (*αhν*)^2^ plotted against (*hν*), the forbidden bandwidth values of the product samples could be estimated. The forbidden bandwidth values of NH_2_-MIL-53(Fe), MIL-53(Fe), MIL-100(Fe), and g-C_3_N_4_, were 1.472 eV, 1.779 eV, 1.979 eV, and 2.759 eV, respectively. According to the relevant literature, it was known that the smaller the value of forbidden bandwidth, the stronger the corresponding light-accepting ability [34]. So, NH_2_-MIL-53(Fe) may possess better light receiving ability. This facilitated the transport of excited electrons in the material under photocatalysis and improved the photocatalytic activity.

The recombination of photogenerated electron–hole pairs in composite photocatalysts could release energy in the form of fluorescence. This would cause the photocorrosion phenomenon. This phenomenon could be measured by steady-state photoluminescence spectroscopy (PL). Therefore, a higher rate of photogenerated carrier recombination means a higher PL fluorescence intensity and lower photocatalytic activity. As shown in Figure 6c, the four materials exhibited noticeable fluorescence signals between 600 nm and 700 nm. A comparison of the PL spectra reveals that they followed the order: g-C_3_N_4_ > MIL-53(Fe) > MIL-100(Fe) > NH_2_-MIL-53(Fe), from strongest to weakest. This also implies that that the photocatalytic activity follows the order: NH_2_-MIL-53(Fe) < MIL-100(Fe) < MIL-53(Fe) < g-C_3_N_4_.

### 2.3. Electrochemical Analysis

#### 2.3.1. AC Impedance Analysis

**Figure 7:**
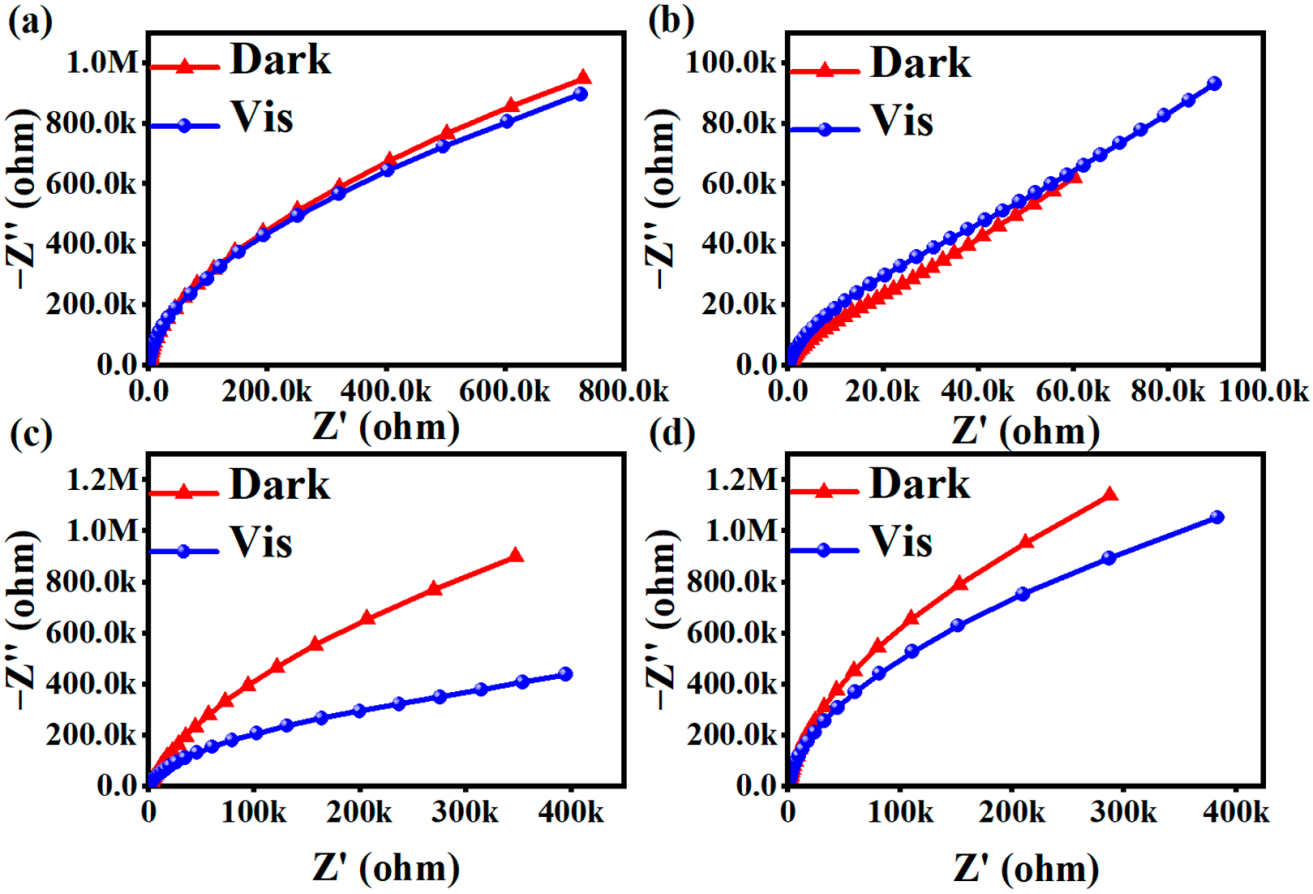
AC impedance diagrams for (**a**) MIL-53(Fe), (**b**) NH_2_-MIL-53(Fe), (**c**) MIL-100(Fe), and (**d**) g-C_3_N_4_.

The photogenerated electron–hole separation efficiency of photocatalysts before and after light exposure is reflected by the AC impedance Nyquist spectra. The size of the arc radius in the electrochemical impedance spectroscopy (EIS) Nyquist spectra reflected the magnitude of the photogenerated electron–hole separation rate and the magnitude of the reaction rate of the photocatalysts. The smaller the arc radius was in the AC impedance spectra, and a larger photogenerated electron–hole separation rate was for the material upon illumination. As shown in Figure 7a, the arc radii of the EIS Nyquist plots measured after its illumination are smaller than the arc radius before illumination, but not obviously so. Figure 7b clearly did not fit this scenario. As shown in Figure 7c,d, the arc radii of the EIS Nyquist plots measured after the illumination of these two groups of products were significantly smaller than those before the illumination. Among them, the change in Figure 7c was the most obvious. These results indicate that the photogenerated electron–hole pairs of MIL-100(Fe) materials were separated at a faster rate, leading to faster charge transfer on their surfaces, which promotes the photocatalytic reaction and enhances the photocatalytic activity.

#### 2.3.2. Photocurrent Analysis

**Figure 8:**
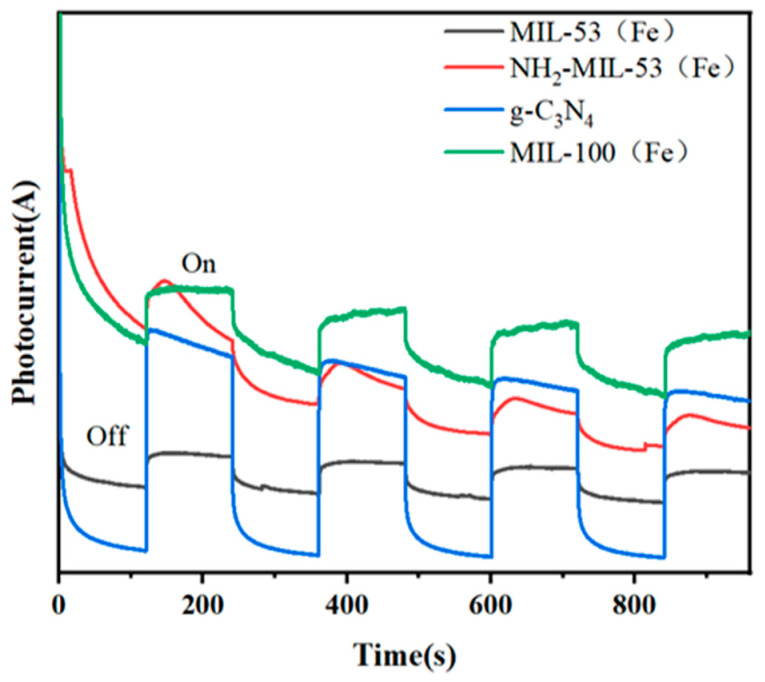
Time–current diagrams for MIL-53(Fe), NH_2_-MIL-53(Fe), MIL-100(Fe), and g-C_3_N_4_.

Figure 8 showed the time–current curves of different materials. When the photocatalyst was irradiated by visible light, electron migration occurred on its surface, leading to the formation of a transient photocurrent. The magnitude of this photocurrent reflects the mobility of the photogenerated electrons of the catalyst. MIL-100(Fe) produces the largest photocurrent, while g-C_3_N_4_ produces the smallest photocurrent under visible light radiation.

### 2.4. Photocatalytic Activities

#### 2.4.1. Photocatalytic Performance of Different Materials

**Figure 9:**
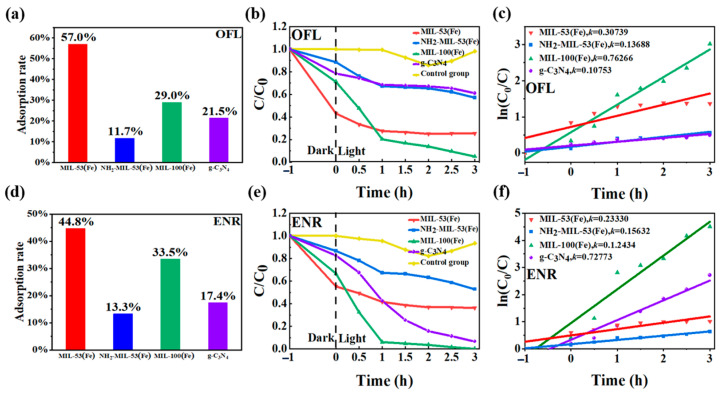
Adsorption efficiencies of (**a**) ofloxacin and (**d**) enrofloxacin on four materials under dark treatment; the degradation efficiencies of (**b**) ofloxacin and (**e**) enrofloxacin under different photocatalytic systems; and variation curves of the reaction rate constant *K* for (**c**) ofloxacin and (**f**) enrofloxacin.

The analyses of the photocatalytic performances of the four materials were carried out. The initial concentrations of both OFL and ENR were set at 10 mg dm^−3^ with 80 mg of the photocatalytic materials added. The samples were taken and examined at 0 h, 1 h, 2 h, and 3 h of light irradiation, respectively. From Figure 9a,d, it could be seen that all four materials adsorbed OFL and ENR to different degrees after dark treatment for 1 h, except for the control group. MIL-53(Fe) and MIL-100(Fe) showed a stronger adsorption performance of up to 30%. The adsorption efficiency of NH_2_-MIL-53(Fe) was worse, with the adsorption rates of OFL and ENR at only 12% and 13%. The reason could be that both MIL-53(Fe) and MIL-100(Fe) feature highly porous structures, offering extensive surface areas for adsorption. The distinct pore geometry and size distribution of these materials enhance the efficient capture and retention of antibiotic molecules. Additionally, the specific surface area of NH_2_-MIL-53(Fe) was only 2.81 m^2^/g, which is far less than that of the other MIL-53(Fe) and MIL-100(Fe). It also exhibits a smaller pore volume due to the H-bonding between the NH_2_ and OH groups in NH_2_-MIL-53(Fe). Thus, MIL-53(Fe) and MIL-100(Fe) show a stronger adsorption performance than g-C_3_N_4_ and NH_2_-MIL-53(Fe). As shown in Figure 9b,e, when the light source was turned on, the material MIL-100(Fe) exhibited the best photocatalytic effect. After 3 h under visible light, the degradation efficiency of OFL reached 95.1%, while enrofloxacin was basically completely degraded. Among other materials, g-C_3_N_4_ showed a better photocatalytic effect on enrofloxacin, which was reduced from 10 mg dm^−3^ to 0.66 mg dm^−3^ with a degradation efficiency of 99.34%. However, it was less effective for OFL, with an efficiency of only 17.9%. The material MIL-53(Fe) mainly showed a better adsorption effect, but the photocatalytic effect was not obvious. After 3 h of photocatalytic effect, the photocatalytic rate for OFL and ENR was only 32% and 9%, respectively, after deducting the adsorption effect. The photocatalytic effect of the material NH_2_-MIL-53(Fe) for OFL and ENR was average, and the effective degradation rates by eliminating the adsorption effect were 31% and 34%.

The degradation rate was another way to evaluate the photocatalytic activity of the product. Figure 9c,f showed the variation curves of the reaction rate constant *K* for the degradation of floxacin by different materials under visible light. According to the Langmuir–Hinshelwood kinetic modification model [35], the larger the value of *K*, the better the photocatalytic performance of the product. The formula was d*c*/d*t* = −*K_c_ c ln*(*c*_0_/*c_i_*) = *K_c_t* (where *K_c_* was the kinetic rate constant of the first-stage reaction). With the *ln*(*c*_0_/*c_i_*) as the vertical coordinate and visible time t as the horizontal coordinate, the kinetic characteristic curve was plotted. The photocatalytic degradation of 10 mg dm^−3^ of floxacin was carried out, and the kinetic curves and parameters obtained after fitting are shown in Table 2. The linear correlation coefficient *R*^2^ of the kinetic equation of MIL-100(Fe) was greater than 0.91. These results indicated that the photocatalytic degradation of floxacin by MIL-100(Fe) conformed to the kinetic equation of the first-order reaction.

#### 2.4.2. Effect of pH on the Degradation of Floxacin

**Figure 10:**
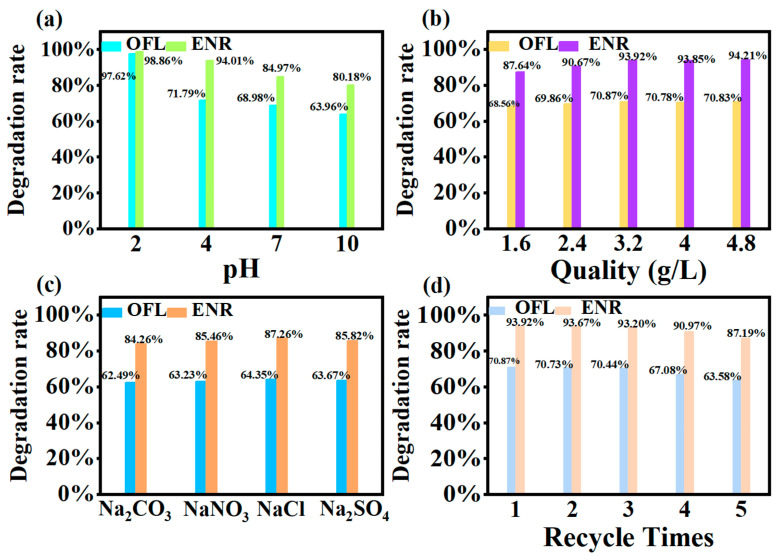
(**a**) Effect of different pH on degradation rate; (**b**) Effect of different inputs’ catalyst quality on degradation rate; (**c**) Effect of ion coexistence on degradation rate; (**d**) Experiment on the number of cycles under the MIL-100(Fe) photocatalytic system OFL and ENR.

The pH was adjusted with 0.01 mol dm^−3^ hydrochloric acid and sodium hydroxide, respectively. As could be seen in Figure 10a, the degradation rate of both kinds of sarsin was the highest in acidic medium, reaching 97.62% and 98.86%, respectively. This was followed by neutral conditions, while the worst results were observed in alkaline conditions of 63.96% and 80.18%. This was due to the fact that, under acidic conditions, floxacin was more easily protonated and positively charged. It promoted effective contact with the surface of the MIL-100(Fe) material and increased the photocatalytic activity. Meanwhile, in the weak acidic environment, H^+^ may participate in the photocatalytic reaction to generate ▪HO_2_ radicals, further promoting the generation of ▪O_2−_ and ▪OH [36].

The isoelectric point of the adsorbent was determined by the salt addition method. The pH (*pH_i_*) was measured by adjusting the pH of 50.0 cm^3^ of electrolyte with 0.01 mol dm^−3^ HNO_3_ and 0.01 mol dm^−3^ NaOH in the range of 2.0–11.0 using NaNO_3_ solution (0.1 mol dm^−3^) as an inert electrolyte. Then, 0.2 g of adsorbent was added to each solution, which was sealed and stirred for 24 h to measure the final pH (*pH_f_*). The *pH_f_* value was plotted against the initial pH value (*pH_i_*) and the pI was determined from the *pH_i_* = *pH_f_* [37]. The isoelectric point (pI) of the synthesised MIL-100(Fe) sorbent determined by the salt addition method was 2.02, which showed that the sorbent was strongly acidic. The sorbent surface was positively charged at pH < 2.02 and negatively charged at pH > 2.02.

#### 2.4.3. Effect of Different Inputs on the Degradation of Floxacin

Figure 10b demonstrates the effect of different addition amounts on the degradation rate. It could be seen that the degradation rate increased with the increase in the photocatalyst addition. And, the degradation rate was basically maintained when the addition reached a certain value. The optimum input amount of photocatalyst MIL-100(Fe) was 3.2 g dm^−3^. When the addition amount was insufficient, the photocatalyst could not provide enough active sites for floxacin, leading to a decrease in catalytic activity. With the addition amount increased, the contact area and irradiation area of the photocatalyst expanded, enhancing catalytic activity. However, beyond 3.2 g dm^−3^, the degradation rate began to level off, indicating that a critical value had been reached. An excessive amount of photocatalyst might have led to aggregation or precipitation, reducing active sites.

#### 2.4.4. Effect of Different Ions on the Degradation of Floxacin

After adding 40 mg dm^−3^ of NaCl, Na_2_CO_3_, Na_2_SO_4_ and NaNO_3_, respectively, it could be found from Figure 10c that the overall degradation rate of both kinds of floxacin decreased. This may be due to the lower concentration of coexisting ions, so the overall effect was small. CO_3_^2−^ has the greatest effect, probably because CO_3_^2−^ acts as a free radical trapping agent, resulting in a significantly lower degradation rate of the treatment. While Na_2_CO_3_ showed a weak alkalinity, an increase in pH was not favourable for degradation. In addition, Cl^−^, SO_4_^2−^ and NO_3_^−^ might compete with floxacin and obscure the active sites of the photocatalysts, thus inhibiting the photocatalytic activity.

#### 2.4.5. Photocatalytic Cycle

Repeatability and stability of photocatalytic materials play an important role in the study. The catalysts after photocatalysis for 3 h were continually used for the next photocatalysis by refreshing the floxacin solution. From Figure 10d, it could be seen that after five rounds of regeneration, the OFL degradation rate could still maintain more than 60%. The ENR degradation rate could still maintain more than 80%. This indicated that the material had a good cyclic regeneration ability [38].

#### 2.4.6. Degradation Pathway

**Figure 11:**
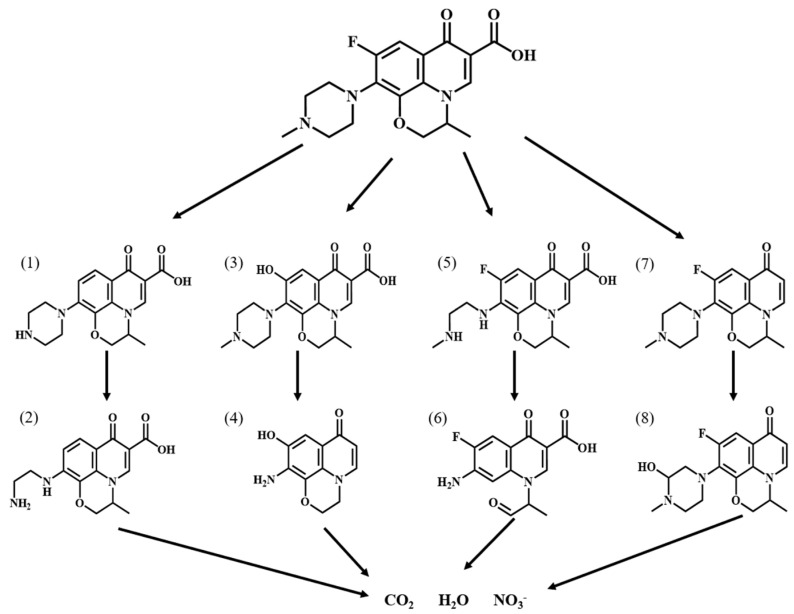
Possible degradation pathways of Ofloxacin by MIL-100(Fe).

**Figure 12:**
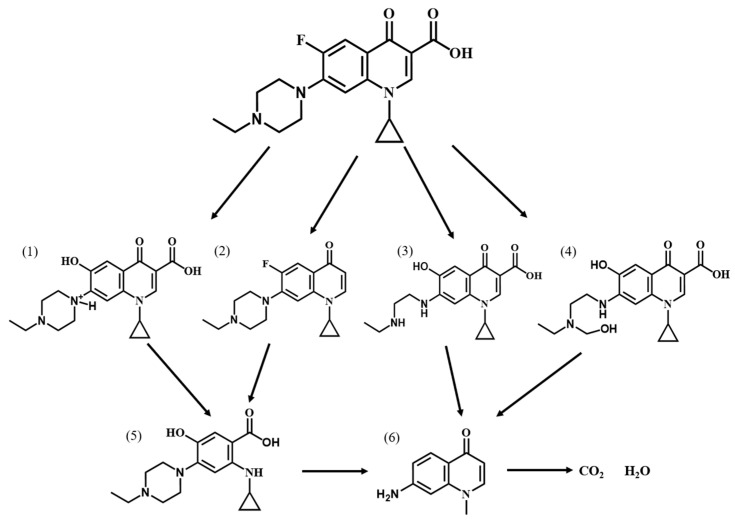
Possible degradation pathways of Enrofloxacin by MIL-100(Fe).

In order to investigate the degradation pathway of Ofloxacin, HPLC-MS was used to identify the organic products. The protonated molecules as well as fragment ions of Ofloxacin were obtained by LC-MS analysis and the possible degradation pathways of Ofloxacin are shown in Figure 11. In the first step, Ofloxacin underwent defluorination and decarboxylation to form (1) and (4), defluorination and oxidation to form (2), and piperazine substituent breakage to form (3). The hydroxyl radicals generated could then attack the piperazine substituent, oxazine substituent and carboxyl group in the structure of Ofloxacin to form (2), (4), (6), and (8). The carboxyl group was attacked by the hydroxyl radical and decarboxylates to produce CO_2_, and the piperazinyl and oxazinyl substituents broke under the attack of the hydroxyl radical to produce a number of small molecular weight carboxylic acids and inorganic molecules such as H_2_O and NO_3_^−^ [39]. As shown in Figure 12, the degradation process of Enrofloxacin was mainly through decarboxylation, defluorination, and oxidation of the piperazine ring to produce six possible intermediate products. Firstly, enrofloxacin underwent defluorination and oxidation to form compound (1), decarboxylation to form (2), and piperazine ring breakage to form (3), (4). Secondly, the piperazine ring cleavage separates and compounds (1), (2) were further attacked by hydroxyl radicals to form product (5). Subsequently, the products (3), (4), (5) underwent decarboxylation, and the ring opening of the cyclopropane and benzene rings and were broken and cleaved to produce smaller organic compounds (6), ultimately generating CO_2_ and H_2_O [40].

Table 3 compared the photocatalytic degradation of pollutants by MIL-100 composites with other semiconducting photocatalysts. It could be seen that the newly synthesised MIL-100 photocatalyst showed a good photodegradation of organic pollutants.

#### 2.4.7. Photocatalytic Reaction Mechanism

**Figure 13:**
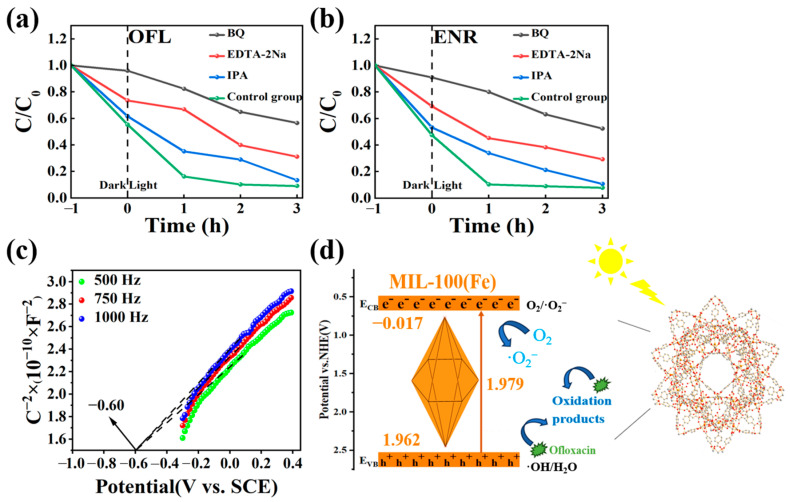
(**a**,**b**) The effect of different free radicals on the degradation rates of OFL and ENR in MIL-100(Fe) photocatalytic system; (**c**) Mott–Schottky curve of MIL-100(Fe) and (**d**) the schematic illustration of possible photocatalytic mechanism of MIL-100(Fe) degradation of OFL.

To study the photocatalytic reaction mechanism of MIL-100(Fe), benzoquinone (BQ), isopropanol (IPA), and disodium ethylenediaminetetraacetic acid salt (EDTA-2Na) were used as trappers for the superoxide radical (▪O_2_^−^), hydroxyl radical (▪OH), and hole (h^+^), respectively. As can be seen from Figure 13a,b, the degradation efficiency was affected by the addition of BQ compared to the control group. Specifically, the degradation rates of OFL and ENR decreased from 96% and 99% to 43% and 45%, respectively. The addition of EDTA-2Na also had an inhibitory effect, and the degradation rates of OFL and ENR were both 65% after 3 h of photocatalysis, which were reduced by 31% and 34%. Compared with BQ and EDTA-2Na, the addition of IPA had less effect on the degradation effect, and the degradation rate was basically unaffected after 3 h of photocatalysis. Therefore, it could be concluded that ▪O_2_^−^ plays the main role in the photocatalytic process, followed by holes (h^+^). For future experiments, the promotion of ▪O_2_^−^ production could be considered. 

The understanding of the energy band structure was also crucial for understanding charge transfer. Thus, Figure 13c showed the Mott–Schottky curve in the dark. By analysing the intercept of the tangent line on the *X* axis, the flat band potential (*E_fb_*) of MIL-100(Fe) was calculated to be −0.60 V (vs. SCE). According to the equation E(vs. NHE) = E(vs. SCE) + 0.051pH + 0.24 [45,46,47], the flat band (*E_fb_*) was 0.083 (vs. NHE). The Mott–Schottky curve of MIL-100(Fe) showed a negative tangent slope indicating the presence of an n-type semiconductor. For n-type semiconductors, the energy of the *E_CB_* was typically 0.1 V lower than *E_fb_*. So, the *E_CB_* value for the MIL-100(Fe) was −0.017 V (vs. NHE). By using the equation *E_g_* = *E_VB_* − *E_CB_* (where *E_g_* represents the band gap obtained from the solid-state UV map), the E_VB_ is therefore 1.962 V (vs. NHE) [48]. Based on the above results, Figure 13d illustrates the photocatalytic reaction mechanism of MIL-100(Fe) [49].

## 3. Experimental Sections

### 3.1. Experimental Materials

All the reagents in this study were of analytic grade and commercially available. Ferric nitrate nonahydrate (Fe(NO_3_)_3_·9H_2_O), iron trichloride hexahydrate (FeCl_3_·6H_2_O), terephthalic-acid (C_8_H_6_O_4_), trimesic acid (H_3_BTC), 2-aminoterephthalic acid (C_8_H_7_NO_4_), melamine (C_3_H_6_N_6_), hydrochloric acid (HCl), ethanol (C_2_H_5_OH), sodium hydroxide (NaOH), DMF (C_3_H_7_NO), acetonitrile (C_2_H_3_N), enrofloxacin (C_19_H_22_FN_3_O_3_), ofloxacin (CHFN_3_O_4_), disodium ethylenediaminetetraacetic acid (EDTA-2Na), isopropanol (C_3_H_8_O), and p-benzoquinone (BQ) were analytical grade and purchased at Shanghai McLean Biochemical Technology Co., Ltd. (Shanghai, China).

### 3.2. Preparation of Photocatalyst

#### 3.2.1. Preparation of MIL-53(Fe)

FeCl_3_·6H_2_O (270 mg, 0.083 mol dm^−3^) and terephthalic acid (166 mg, 0.083 mol dm^−3^) were dissolved in 12 cm^3^ of DMF. Ultrasonic dispersion was performed for 15 min. The resulting solution was transferred to the Teflon reactor and subjected to a reaction at 150 °C for 15 h. Then, centrifuged the solution at 6000 r/min and washed the obtained solid with DMF for 3–5 times. Finally, dried the product in a 150 °C oven for 12 h.

#### 3.2.2. Preparation of NH_2_-MIL-53(Fe)

FeCl_3_·6H_2_O (270 mg, 0.1 mol dm^−3^) and 2-aminoterephthalic acid (181 mg, 0.1 mol dm^−3^) were added into 10 cm^3^ of DMF, and the mixture was stirred at room temperature for 10 min. The well-mixed solution was then transferred to the PTFE-lined reactor and heated to 150 °C for 6 h. After cooling to room temperature, the sample was separated by centrifugation at a rate of 6000 r/min for 4–6 cycles. The sample was then alternately washed with ethanol and deionised water. Finally, the sample was dried in a blast drying oven at 150 °C for 12 h.

#### 3.2.3. Preparation of MIL-100(Fe)

**Scheme 1:**
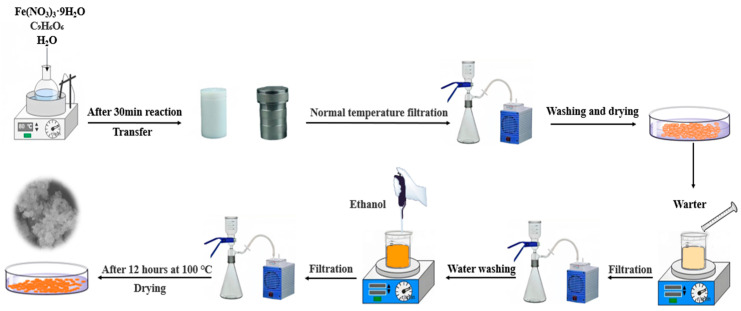
Illustration of the preparation processes of MIL-100(Fe).

Scheme 1 showed the synthetic procedure of MIL-100(Fe) composites [50]. Fe(NO_3_)_3_·9H_2_O (2.02 g, 0.016 mol dm^−3^), trimesic-acid (0.7 g, 0.011 mol dm^−3^), and H_2_O (30 cm^3^) were added into a beaker and mixed homogeneously, and then stirred vigorously for 30 min at room temperature, the ratio of the three was 1:0.66:35. Then, the mixed solution was transferred into the Teflon reactor and heated to 95 °C for 12 h. After cooling to room temperature and vacuum filtration, the product was washed with sufficient deionised water and dried in an oven at 60 °C for 5 h. After drying, the product was stirred with 200 cm^3^ of H_2_O at 80 °C for 5 h. The mixture was filtered and dried. Adding 20 cm^3^ of ethanol into the product with stirring at 60 °C for 3 h. After filtration, the sample was washed alternately with ethanol and deionised water for 3–5 times, and finally dried in an oven at 100 °C for 12 h.

#### 3.2.4. Preparation of g-C_3_N_4_

Five grams of melamine was put into a crucible and calcined in a muffle furnace with a controlled heating rate of 5 °C/min at 550 °C for 4 h. After naturally cooling to room temperature, the product was taken out and grinded to a fine powder with an onyx mortar to obtain a yellow powdery product.

### 3.3. Characterisation of Photocatalysts

The synthesised and modified photocatalysts were thoroughly characterised using a variety of techniques. The morphology and the microscopic features of the specimens were analysed by S-4800 electron microscope (SEM) from the Hitachi, Tokyo, Japan. The crystalline structure of the specimens was determined using the D8 Advance X-ray diffractometer (XRD) from the Bruker (Karlsruhe, Germany) with Cu Ka radiation (40 kV, 100 mA, λ = 0.15406 nm). The composition of the material was tested using IRAffinity-1 Fourier transform infrared spectrometer (IR) from the company Shimadzu (Kyoto, Japan). Adsorption–desorption curves were determined using an ASAP 2020HD88 specific surface areas (BET) under nitrogen. Ultraviolet–visible (UV–vis) spectra recorded by a U-4100 instrument from the Japanese company Hitachi. Photoluminescence spectra were tested with a CARY ECLIPSE fluorescence spectrophotometer (PL) from the American company Agilent Corporation (Santa Clara, CA, USA). The HPLC-MS was tested using the Synapt G2-Si HDMS from Waters (Milford, MA, USA).

### 3.4. Electrochemical Tests

The Nyquist impedance spectrum (AC impedance), transient photocurrent, and Mott–Schottky curves were tested using a CHI760E Electrochemical Station (Chenhua, Shanghai, China) in constant potential mode with a 350 W xenon lamp as the light source. The platinum wire was used as the counter electrode and a saturated calomel was used as the reference electrode. The working electrode was fabricated as follows: 30 mg of photocatalyst was weighed and mixed with 2 cm^3^ of ethanol and 200 µL 5.0% Nafion solution, followed by sonication for 50 min to disperse them. Next, 150 µL of the catalyst solution was dispensed onto the pretreated F-doped tin oxide (FTO) glass electrode using a pipette. The resulting FTO glass electrode was dried at room temperature to form the electrode [51].

### 3.5. Photocatalytic Tests

The 3.2 g dm^−3^ of MIL-53(Fe), NH_2_-MIL-53(Fe), MIL-100(Fe), and g-C_3_N_4_ were added into 25 cm^3^ of enrofloxacin and ofloxacin solution with concentration of 10 mg dm^−3^, respectively. The enrofloxacin and ofloxacin solution without catalysts was used as the control group.

Considering the potential adsorption properties of the designed materials, the five sets of solutions were first put into the feeding box wrapped with tinfoil for dark treatment for 1 h, so that the targets could be effectively adsorbed on the materials, first to prevent experimental errors caused by the influence of light. Then, put them into the photocatalytic apparatus. Using the 500 W ultraviolet mercury lamp (Beijing Zhongjiao Jinyuan Technology Co., Ltd, Beijing, China) as the photocatalytic apparatus, the light was converted into visible light by inserting a filter. Then, sample solutions were stirred under visible light for 3 h. The sample solutions after 0 h, 1 h, 2 h, and 3 h of light irradiation were taken using a disposable syringe. They were then filtered by using the MCE (mixed cellulose) aqueous filter with a pore diameter of 0.22 μm and stored in a 2 cm^3^ injection bottle. The control group was treated in the same way. The concentrations of enrofloxacin and ofloxacin in the water samples were determined using liquid chromatography. Prior to injection, the autosampling needle was cleaned with acetonitrile solution and lubricated with the samples to be measured. The adsorption *q_t_* (mg g^−1^), removal rate RE (%) and mean relative deviation (MRD) (%) of the photocatalytic material were calculated by the following Equations (1)–(3) [52]:

(1) $$q_{t}=\frac{c_{0}-c_{t}}{m}\cdot V$$

(2) $$\mathrm{RE}\;(\%)=\frac{c_{0}-c_{t}}{c_{0}}\cdot 100$$

(3) $$\mathrm{MRD}\;(\%)=\frac{\sum_{i=1}^{n}\left|q_{i,\exp}-q_{i,\mathrm{cal}}\right|}{\bar{q_{i,\exp}}\cdot n}\cdot 100$$

where *c*_0_ and *c_t_* were the initial concentration of floxacin and the concentration at different times (mg dm^−3^), *m* was the mass of the adsorbent (g), *V* was the volume of the solution, *q_i,exp_* (mg g^−1^) was the amount of adsorption measured at the experimental point *i*, *q_i,cal_* (mg g^−1^) was the amount of adsorption at the experimental point *i* calculated by the kinetic modeling; and *n* was the number of experimental points.

### 3.6. Photocatalytic Degradation Mechanism

In total, 250 μL of OFL and ENR solution at a concentration of 1000 mg dm^−3^ was added into a 25 cm^3^ cuvette, fixed and configured to a concentration of 10 mg dm^−3^, along with a standard solution (10 mg dm^−3^ of enrofloxacin and ofloxacin solution). Four cuvettes were taken and 3.2 g dm^−3^ of MIL-100 (Fe) was added to each of them. Three of the tubes were filled with 0.004 mol dm^−3^ of benzoquinone (BQ), isopropanol (IPA), and disodium ethylenediaminetetraacetic acid salt (EDTA-2Na) as the trapping agents of the superoxide radical (▪O_2_^−^), hydroxyl radical (▪OH) and hole (h^+^), respectively, and the other group was the control group.

### 3.7. Liquid Chromatography Analysis Conditions

The concentration of OFL and ENR were measured by liquid chromatography. The mobile phases were a mixture of 0.2% acetic acid solution and acetonitrile with 4:1 weight ration; the column model was Athena C18, 4.6 × 250 mm, 5 μm; 50 μL was injected into each needle; the absorption wavelength of the UV detector was set at 275 nm; the temperature of the column was 30 °C; the flow rate was 1.0 cm^3^/min; and the analytical time was 10 min. The data were comparatively recorded and analysed. The peak time and peak area were observed. Concentrations were calculated for each time period based on the relationship between liquid chromatography peak area and concentration being proportional to each other.

## 4. Conclusions

In conclusion, four photocatalytic materials, MIL-53(Fe), NH_2_-MIL-53(Fe), MIL-100(Fe), and g-C_3_N_4_, were prepared by hydrothermal synthesis and applied to the removal of ofloxacin and enrofloxacin. The MIL-100(Fe) material exhibited the best photocatalytic effect with the degradation efficiency of ofloxacin reached 95.1% under visible light, and enrofloxacin was basically completely degraded. The effects of pH, inputs and different ions on the degradation of floxacin by MIL-100(Fe) material were investigated. Moreover, the exploration of the photocatalytic mechanism was studied and summarised as follows:

1. Fluorescence and UV diffuse reflectance analyses of the four materials showed that the photogenerated carrier separation efficiency of NH_2_-MIL-53(Fe) was the best, followed by MIL-100(Fe).
2. According to electrochemical tests on the four materials, the MIL-100(Fe) materials had a large separation rate of photogenerated electron–hole pairs. The time–current curves indicated that MIL-100(Fe) produced the largest photocurrent and the best photocatalytic performance.
3. By comparing the photocatalytic degradation effects of four materials, it was found that MIL-100(Fe) had the best catalytic ability in degrading the ofloxacin and enrofloxacin. The degradation effect was best under acidic conditions. The addition of other ions did not affect the degradation rate to a great extent, the optimal amount of the photocatalyst added was 0.08 g. The degradation on floxacin by MIL-100(Fe) exhibited a good cycling performance.
4. The ▪O_2_^−^ played a major role in the photocatalytic reaction process, followed by holes (h^+^).

## Figures and Tables

**Table 1:** BET date of MIL-53(Fe), NH_2_-MIL-53(Fe), MIL-100(Fe) and g-C_3_N_4._

Material	*S_BET_* m^2^/g	*V_pore_* cm^3^/g	Pore Size/nm
MIL-53(Fe)	17.04	0.02	4.73
NH_2_-MIL-53(Fe)	2.81	0.01	18.68
MIL-100(Fe)	900.15	0.45	1.98
g-C_3_N_4_	52.72	0.19	14.05

**Table 2:** Characteristic values of photocatalytic reaction kinetics for MIL-53(Fe), NH_2_-MIL-53(Fe), MIL-100(Fe), and g-C_3_N_4_.

**Photocatalytic reaction kinetic eigenvalues for degradation of OFL**				
**Materials**	***K***	**First-order kinetic equations**	***R*** **^2^**	**MRD (%)**
MIL-53(Fe)	0.32832	ln(*c*_0_/*c*) = 0.32832*t* + 0.64929	0.77501	7.44271
NH_2_-MIL-53(Fe)	0.14225	ln(*c*_0_/*c*) = 0.14225*t* + 0.15974	0.94451	9.14266
MIL-100(Fe)	0.76772	ln(*c*_0_/*c*) = 0.76772*t* + 0.62241	0.96997	6.64729
g-C_3_N_4_	0.11488	ln(*c*_0_/*c*) = 0.11488*t* + 0.18819	0.89390	8.63929
**Photocatalytic reaction kinetic eigenvalues for degradation of ENR**				
**Materials**	***K***	**First-order kinetic equations**	***R*** **^2^**	**MRD (%)**
MIL-53(Fe)	0.20978	ln(*c*_0_/*c*) = 0.20978*t* + 0.42368	0.77755	8.04445
NH_2_-MIL-53(Fe)	0.12618	ln(*c*_0_/*c*) = 0.12618*t* + 0.13871	0.85743	4.61381
MIL-100(Fe)	1.17273	ln(*c*_0_/*c*) = 1.17273*t* + 0.98767	0.89821	8.10197
g-C_3_N_4_	0.99892	ln(*c*_0_/*c*) = 0.99892*t* + 0.53164	0.80522	8.99683

**Table 3:** Comparative study of MIL-100 composites with other photocatalysts.

Catalysts (Weight)	Pollutant Concentration	Time (min)	Degradation Rate (%)	Refs.
Sm_2_(WO_4_)_3_@g-C_3_N_4_ (50 mg)	MP (20 mg dm^−3^)	80	97.00	[41]
	Ofloxacin (20 mg dm^−3^)	40	98.00	
CDs/ZnO-H400 (50 mg)	RR141 (10 mg dm^−3^)	240	98.00	[42]
	Ofloxacin (10 mg dm^−3^)		99.00	
C-dopedZnO (0.05 g)	MB (10 mg dm^−3^)	180	92.00	[43]
	Ofloxacin (15 mg dm^−3^)		93.00	
CoAl-LDH (150 mg)	Enrofloxacin (10 mg dm^−3^)	60	97.72	[44]
MIL-100 (0.08 g)	Ofloxacin (10 mg dm^−3^)	180	95.10	This work
	Enrofloxacin (10 mg dm^−3^)		99.00	

## Data Availability

Data are contained within the article.
